# Precision Agriculture Using Soil Sensor Driven Machine Learning for Smart Strawberry Production

**DOI:** 10.3390/s23042247

**Published:** 2023-02-16

**Authors:** Rania Elashmawy, Ismail Uysal

**Affiliations:** Department of Electrical Engineering, University of South Florida, 4220 East Fowler Avenue, Tampa, FL 33620, USA

**Keywords:** smart agriculture, machine learning, IoT, harvest forecasting, sustainable farming, food quality prediction

## Abstract

Ubiquitous sensor networks collecting real-time data have been adopted in many industrial settings. This paper describes the second stage of an end-to-end system integrating modern hardware and software tools for precise monitoring and control of soil conditions. In the proposed framework, the data are collected by the sensor network distributed in the soil of a commercial strawberry farm to infer the ultimate physicochemical characteristics of the fruit at the point of harvest around the sensor locations. Empirical and statistical models are jointly investigated in the form of neural networks and Gaussian process regression models to predict the most significant physicochemical qualities of strawberry. Color, for instance, either by itself or when combined with the soluble solids content (sweetness), can be predicted within as little as 9% and 14% of their expected range of values, respectively. This level of accuracy will ultimately enable the implementation of the next phase in controlling the soil conditions where data-driven quality and resource-use trade-offs can be realized for sustainable and high-quality strawberry production.

## 1. Introduction

In the world of big data sustained by ubiquitous applications of sensor networks, artificial intelligence (AI) and machine learning (ML) create significant opportunities in multidisciplinary fields, including healthcare, financial services, and smart agriculture. According to the U.S. Department of Agriculture (USDA), the agriculture field and surrounding industries contributed USD 1.109 trillion to the U.S. gross domestic product and provided 10.9% of the total employment in 2019 [1,2]. This paper introduces a holistic approach, which includes a sensor network with real-time connectivity to the cloud, algorithm-driven controllers, and advanced machine learning algorithms to enable a fully automated soil conditioning system. This paper aims to improve the sustainability of strawberry production, which can easily be scaled to other kinds of special crops. Smart farming systems use sensory data and real-time monitoring devices to acquire high-resolution data from the field and the environment surrounding the crop to improve the crop quality and quantity while decreasing cost and increasing sustainability.

Researchers have focused on the yield prediction sector to support the economy and develop a sustainable supply across many different crops. Strawberry fruit is an essential crop for the U.S. economy, as the U.S. is the second-largest strawberry producer worldwide, contributing to 26.2% of the world’s production with 231 tons in 2017–2018 [3]. The importance of the strawberry crop worldwide provided the motivation to study the effects of the natural environment and soil physical characteristics on the quality of the strawberry, and not just the yield. One of the major factors that has been continuously investigated through different studies is the water content and its effects on the yield and strawberry quality [4,5]. Strawberry cultivars have different demands of water content and fertilization to reach acceptable levels of both quality and yield [4,5,6,7,8,9,10]. Previous literature reports that a water deficit has a drastic effect on the strawberry yield and marketability (size of the fruit) on most strawberry cultivates [4,7]. At the same time, the overuse of water increases the cost and subsequently reduces the water resources for other areas [11]. The drip irrigation system for strawberry is an efficient way to save 50% more water compared to the overhead sprinklers [12]. Furthermore, the overuse or wrong kind of fertilization can harm the strawberry size and sweetness [8,13]. Wu et al. [10] studied the effect of four different factors (nitrogen (N), phosphorus (P), potassium (K), and water) on the yield and the strawberry sweetness ratio (SSC/TTA), where they found a significant effect on the yield and SSC when they applied the N fertilizer. While the water content had a significant effect on yield, P fertilizers had a significant effect on the sweetness ratio. However, they noticed a decline in the yield, sweetness ratio, and firmness when they applied fertilizer and water extravagantly [10,14]. The sweetness ratio and the development of the strawberry cultivars are reflected in the ripening stage of the fruit, which affects the strawberry color [15]. The combination of all the factors, including water content and fertilizer levels, with optimal irrigation schedule and techniques, still represent an open field for research. As the new technologies help explore and adjust these combinations, machine learning and data mining help improve the agricultural process for applications including crop prediction, disease, and weed detection, and species recognition [16].

**Figure 1:**
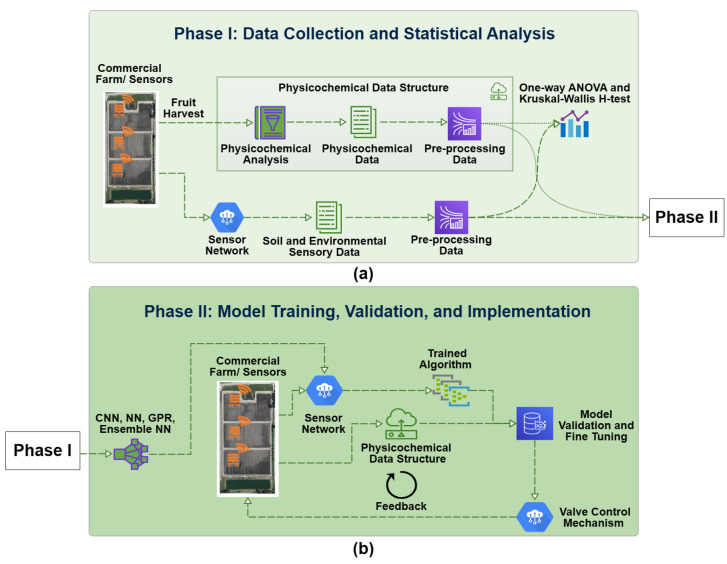
The proposed framework with two stages. (**a**) The first stage is data collection from the field, data pre-processing, and model training. (**b**) The second stage is testing the developed models with new field data and implementing the control hardware based on the model predictions.

Over the past decade, most of the research (authors of this paper included) focused on evaluating the quality of strawberries postharvest with little attention to how to control the quality of the crop pre-harvest. This study proposes a holistic system, which is comprised of multiple components in the form of a wireless sensor network to collect real-time high-resolution data from the soil, machine learning algorithms to predict potential harvest quality from the measured data and a control system to adjust soil parameters, including water content (WC) and electrical conductivity (EC) to achieve desired physicochemical characteristics at the point and time of harvest. The high-resolution data which match the soil measurements with the postharvest quality analysis has been presented in [17]. As the follow-up publication, this paper will focus on the data inference part of the project using machine learning tools, including both empirical (neural networks, ensembles of neural networks (ENN)) and statistical (Gaussian Process Regression (GPR)) approaches. Figure 1 shows the proposed system in full, where the general aim is to regulate the soil water and fertilizer levels both in real-time and on-demand for optimal physicochemical qualities at the harvest. The study’s results so far prescribe several steps that can be taken to enhance strawberry cultivation through soil sensor-based real-time field monitoring. For instance, the control algorithm can be tested in a field where water levels can be monitored and adjusted using real-time smart controllers at drip line valves, thus optimizing resource utilization based on dynamic environmental and weather factors. This article will focus on the data collection and model training, which are required to ultimately control the smart valves at each drip line.

Previous studies have shown less than stellar results regarding strawberries’ overall sensory qualities, which do not always satisfy the customers [18]. As was also shown in the literature, local area information has a significant effect on the model’s accuracy [19,20,21]. This provides a strong incentive to find the optimal pre-harvest conditions for different cultivars and to develop a robust algorithm to determine the relationship between the local soil properties and the strawberry’s physicochemical characteristics.

## 2. Materials and Methods

**Figure 2:**
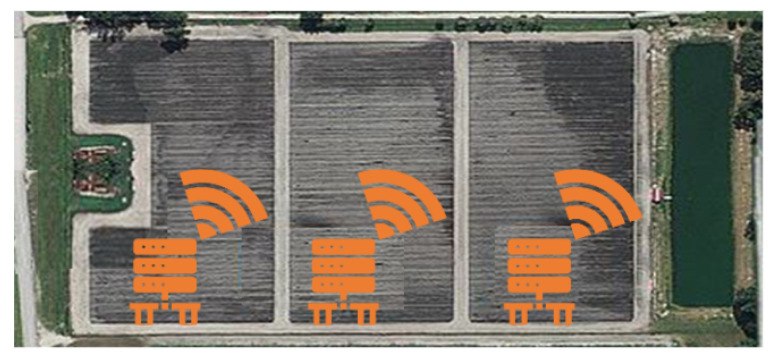
The satellite view of the farm in Plant City, Florida, where the real-time data loggers were placed, and the commercial harvest was performed.

This study was conducted using data collected from an approximately 40 acre commercial strawberry farm in Plant City, Florida. The reader can learn more about the data collection process and the statistical analysis of the data in the first publication of this study [17]. As shown in Figure 2, a bird’s-eye view is provided for the farm with three time-series data loggers from three different regions, each with two terminals connected to three sensors. The markers in Figure 2 represent the location of each logger, which were strategically placed to sample from areas of the field with high, medium, and low humidity levels based on natural elevation. The sensors measured the soil water content (m${}^{3}$/m${}^{3}$), electrical conductivity (mS/cm), and soil temperature (${}^{\circ }$C), for the whole of four months during the 2018–2019 Florida Brilliance strawberry season [22]. The precipitation rate (inch /day) data for Plant City, Florida, were included in this study [23] for that Florida strawberry season in order to see the effect of rain on the water content (WC) and electrical conductivity (EC). There is a relationship between the soil water content (WC), the soil conductivity (EC), and the precipitation rate, similar to what has been found in the literature [17,24]. There is a correlation between the WC level with rainwater, WC level with EC level, and EC level with rainwater where the rainwater obviously increases the WC along with the ions and minerals in the soil, which is then reflected in the EC level, as reported by Friedman et al. [24]. The soil properties such as temperature and WC are significant factors in crop quality and quantity [16,19,25], but since Florida Brilliance Strawberries were grown on an open field in a plastic-covered bed, temperature control is not feasible. Based on the results of the preliminary analysis of the data and what was previously reported in the literature [17,24], the WC and EC were selected as the primary factors in training the ML algorithms.

The dataset includes data points for four harvests during the four months of the 2018–2019 strawberry season, where six different points in the field were harvested to yield a total of 24 multivariate data points after pre-processing the dataset, as shown in Table 1. The 24 multivariate data points include the soil measurements and the corresponding fruit sensory qualities recorded in a collaborating lab at the University of Florida. For each of the 24 samples, the sensory qualities of the harvested strawberries used in the study as output predictions include color and soluble solids content (SSC). Color is an attractive element in strawberry evaluation [18,26] and the basic external quality factor for the postharvest uses of the fruit, as well as a natural indicator of fruit ripeness [15,18,27]. The color of strawberries was analyzed using a machine vision system [28] consisting of a light box, a Nikon D200 digital color camera, and a computer. The camera settings were set to 36 mm focal length, ISO 100 sensitivity, 1/3 s F/11 shutter speed, −1 eV exposure compensation, and direct sunlight white balance. The system was calibrated using a standard red plate from Labsphere. A software program was used to capture images and calculate the average L*, a*, and b* values of the strawberry surface based on five replicates [17]. The measure of redness, “a*”, was taken on the red/green coordinate and considered the definitive indicator of strawberry color in this study. On the other hand, SSC is an indicator of sugar content for sweetness, another primary consumer preferred attribute. SSC was conducted on the homogenized strawberry samples from each harvest. The samples were blended in a high-speed blender for 5 min, then centrifuged at 5000× *g* for 20 min to obtain a clear juice, which was filtered through cheesecloth [17]. The SSC of the resulting supernatant was determined using a Reicher handheld digital refractometer at room temperature [17]. Ultimately, color and SSC was highlighted as the consumer and food industry’s main focus for good strawberry quality [29,30]. There is a correlation between an increase in sugar content in the strawberry as the fruit ripens [31,32]. Furthermore, previous studies have reported the quality of a strawberry is at the peak around day 28 from the fruit set [15], so a 28-day postharvest time window was determined for this study as well.

### 2.1. Machine Learning Methods

The effectiveness of various machine learning algorithms has been proven in many agricultural applications [16,19,20,21,33,34,35]. The overall framework proposed in this study takes inspiration from the fact that the causality between sensory variables and product quality can be used to develop smart control systems for better quality and sustainability as shown in Figure 1. The first phase of the project involved the creation of the dataset from an operational commercial strawberry farm in Central Florida, which included significant collaborations between the stakeholders including the farm and two collaborating academic institutions. Once the data were collected, they were pre-processed to group the recordings within a 4-week time window prior to each harvest on account of the studies in the literature, which discuss how the quality of a strawberry is at its peak between 28–36 days from the fruit set [15]. After some statistical preliminary analysis [17], which demonstrated non-linear relationships between the primary input predictors WC and EC, and the primary quality attributes SSC and color, two scenarios were considered in this study. In the first scenario, the WC was used as the primary input feature to predict associated strawberry qualities, color and soluble solids content (SSC) as the model outputs. In the second scenario, the same outputs were predicted using both WC and EC as joint input features.

The dataset, even though it was collected over a 4-month period with thousands of data points for sensory recordings, still has a challenging size, in light of the limited harvesting capabilities, which allowed for 4 separate occasions for the harvest on the commercial farms. As a result, the dataset has 24 data points and a relatively large feature vector, so it is extremely important to apply the fail-safe measures to prevent the network from over-fitting, such as early stopping and proper cross-validation. Leave-one-out cross-validation was applied in training the algorithms, as described below.

**Figure 3:**
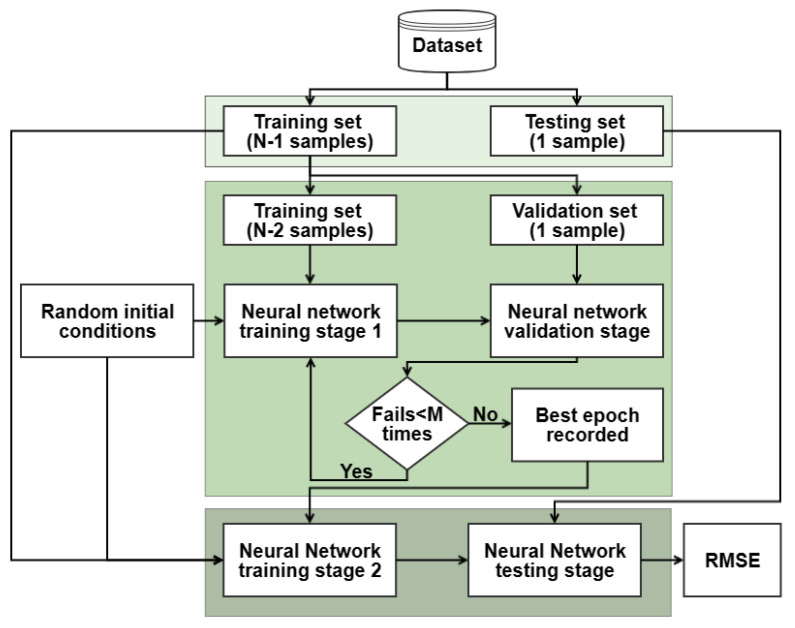
Methodology flow chart used in two-stage neural network training.

One of the recently introduced strategies used in training the empirical models is splitting training data into two partitions, where the first partition is used to train model parameters and the second partition is used to calculate model accuracy at each step. As illustrated in Figure 3, two neural network frameworks were employed in the form of shallow and ensemble (ENN) neural networks, the first to tune the early stopping condition and the second to train and test the dataset. Once the early stopping conditions are reached (i.e., the accuracy on the validation partition starts to drop for N number of consecutive times), the training is stopped, the epoch number is recorded, and the two partitions are combined. The neural network is then trained from scratch using the same initial parameters for the same number of epochs using the entire training set. The final accuracy is reported on the test set, which is not included in any stage of the training process and is alternated between cross-validations.

#### 2.1.1. Empirical Methods

This study includes a thorough comparison between different machine learning topologies which fall under two main categories: empirical and statistical. The first step in this study compares the results of a neural network (NN) to avoid over-fitting on a small dataset to an ensemble of neural networks (ENN), which helps build a more stable algorithm at the expense of error rate.

For each run, leave-one-out cross-validation was used with 23 samples for training and 1 sample for testing. The root mean square error (RMSE) and prediction error percentage (PEP) metrics were used to measure the models’ performance for both frameworks. The PEP is defined as the root mean square error of the network over the quality average (QA) for each feature to provide a general understanding of the relative accuracy with respect to quantifiable metrics for each output, as shown in Equation (1) below. The limitations of the dataset size have ultimately determined the cross-validation size.

(1) $$PEP=\frac{\sqrt{\frac{\sum_{i=1}^{N}{\left(y_{i}-{\hat{y}}_{i}\right)}^{2}}{N}}}{QA}*100$$

Neural Network Data Representation: Different topologies were implemented in the form of one or two hidden layers, with different numbers of neurons in each layer, for an exhaustive analysis with all possible combinations. The input in the first scenario was presented as the WC averages for the 4 weeks prior to each harvest, whereas in the second scenario, the inputs were the WC and EC averages for the 4 weeks prior to each harvest. In both cases, the output was different for each experiment depending on whether univariate or multivariate scenarios were used, such as predicting color separately, or color and SSC together. Each experiment was conducted 50 times for statistical redundancy and the average RMSE on the training and testing sets were recorded. The networks used hyperbolic tangent functions for activation and RMSE as the cost function, whereas PEP was used to evaluate the network prediction error.

Ensemble of Neural Networks: ENN is an enhanced form of a singular neural network, where an ensemble of networks is used with different initial parameters to stabilize the prediction. ENN was used to deal with the randomness of each model and to improve the stability of prediction across all the models, especially considering the size of the dataset. Combining ensembles of neural networks with leave-one-out cross-validation gave us an unbiased and reliable estimation for the model performance and generalized the error [36]. Each experiment was conducted 50 times for statistical redundancy and recorded the average RMSE on the training and testing set. The networks use hyperbolic tangent functions for activation and RMSE as the cost function, whereas PEP was used to evaluate the network prediction error.

#### 2.1.2. Statistical Method

A robust and statistical model was added to corroborate this study—the Gaussian Process Regression (GPR), which has gained immense popularity in recent years—to explore the value of a statistical approach. GPR is a powerful probabilistic model to deal with noisy and small datasets [37], gives us well-calibrated probabilistic outputs, and provides the prediction with its confidence interval.

Gaussian Process Regression: GPR is a non-parametric probabilistic model which uses the Bayesian approach to give a probabilistic output. It uses independent variables to determine the uncertainty in the model prediction and improve the model selection [38]. GPR uses the mean of the input data$,\;m\;\left(X\;∼\;R^{d}\right);$ in the first case, the input data were the WC averages, and in the second case, the input data were the WC and EC averages of the 4-week time scales prior to each harvest and the covariance kernel matrix, $k\left(x,\;x^{{}^{\prime }}\right)$, to calculate the prior probability distribution and predict the posterior probability distribution. GPR maximizes the likelihood of *Y* given *X*, while$\;\epsilon \;∼\;N\;\left(0,\;\sigma_{xx}^{2}\right)\;$ is the noise associated with the data with a zero mean and variance $\sigma_{xx}^{2}$.

(2) $$f\left(x\right)∼GP(m\left(x\right),k\left(x,x^{\prime }\right))$$

(3) $$k\left(x,x^{\prime }\right)=\sigma^{2}exp\left(-\frac{d{\left(x,x^{\prime }\right)}^{2}}{2l^{2}}\right)$$

(4) Y=f(x)+ϵ

Working with GPR on a small dataset helps in saving resources, while computing the kernel matrix as the model complexity, $\left(n^{3}\right)$, is tied to the data dimension [37,38,39]. GPR is still sensitive to over-fitting with a small dataset, so the squared exponential (SE) kernel was used to calculate the prior in our experiments, while adding white noise to overcome the model over-fitting, as described in literature [40,41].

The leave-one-out cross-validation technique was applied to improve the performance and avoid over-fitting, where 23 samples were used for training and one sample for testing. The input–output pairs followed the same pattern as before for each experiment, depending on whether univariate or multivariate scenarios were used, such as predicting color separately or color and SSC together. RMSE was used to measure the models’ performance for both the univariate and multivariate responses, whereas PEP was used to evaluate the network prediction error.

## 3. Results

**Figure 4:**
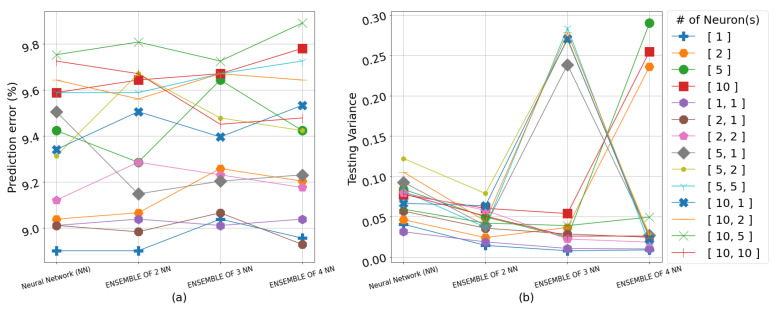
(**a**) Prediction error percentage across 50 experiments for all empirical methods in predicting color from WC. (**b**) The variance across 50 experiments for all empirical methods in predicting color from WC. The legend indicates the number of layer(s) (one or two) in each network and number of neurons(s) in each layer(s).

**Figure 5:**
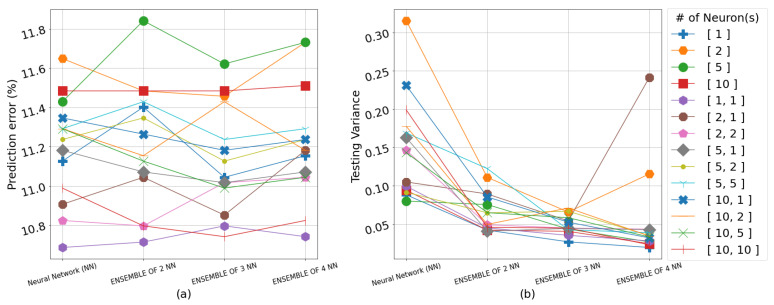
(**a**) Prediction error percentage across 50 experiments for all empirical methods in predicting color from WC and EC. (**b**) The variance across 50 experiments for all empirical methods in predicting color from WC and EC. The legend indicates the number of layer(s) (one or two) in each network and number of neurons(s) in each layer(s).

**Figure 6:**
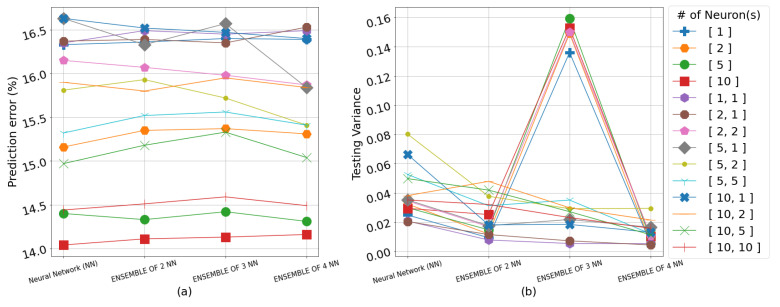
(**a**) Prediction error percentage across 50 experiments for all empirical methods in predicting color and SSC from WC. (**b**) The variance across 50 experiments for all empirical methods in predicting color and SSC from WC. The legend indicates the number of layer(s) (one or two) in each network and number of neurons(s) in each layer(s).

**Figure 7:**
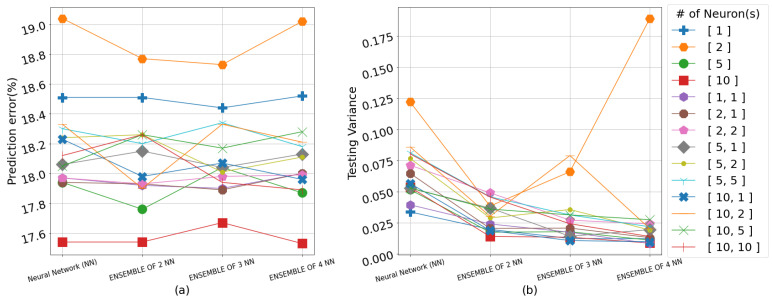
(**a**) Prediction error percentage across 50 experiments for all empirical methods in predicting color and SSC from WC and EC. (**b**) The variance across 50 experiments for all empirical methods in predicting color and SSC from WC and EC. The legend indicates the number of layer(s) (one or two) in each network and number of neurons(s) in each layer(s).

The results overall suggest that the color of a strawberry can be best predicted from the water content (WC) of the soil averaged across the 4 weeks immediately preceding the harvest. Including the electrical conductivity (EC) in the input space for predicting both color and sugar content simultaneously generally resulted in higher RMSE values. The RMSE results on the testing set for all neural network topologies, including singular and ensemble frameworks as well as the GPR models, are shown in detail in Table 2, Table 3 and Table 4. The algorithms use the input representation in the form of weekly averages for WC or the weekly averages for WC and EC, 4 weeks prior to each harvest, whereas Figure 4, Figure 5, Figure 6 and Figure 7 show the prediction error percentage (PEP) and variance across 50 experiments for all the empirical methods for visual illustration. The testing RMSE for each experiment is calculated via leave-one-out cross-validation due to the limited size of the dataset. The mean of RMSE, PEP (error%), and variance of the RMSE for 50 independent runs for each experiment are presented for the empirical methods. Where the mean RMSE indicates the overall performance of a specific algorithm, PEP indicates the prediction error range, and the variance can be used as an indicator of robustness in real-life applications.

### 3.1. Color

**Figure 8:**
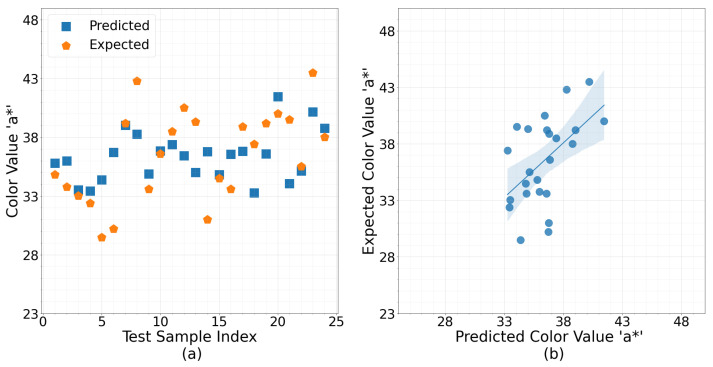
(**a**) Predicted versus observed color values when using the water content as the input to the most accurate NN implementation. (**b**) The relationship between the predicted and observed color pairs for the same implementation.

The performances of the applied topologies for the univariate response, color, support the hypothesis that an empirical tune on an NN with a small dataset can overfit, particularly with sophisticated models, as shown in Table 1. For instance, in some cases, when the number of layers and the number of neurons in each layer increased, the testing RMSE also increased. Moreover, in most cases, the variance decreased as the number of networks increased in the ensemble, as shown in Figure 5b, which means an ensemble could provide more robust performance with a trade-off in mean RMSE. The color prediction results shown in Table 2 display that the lowest RMSE is 3.24 and the PEP is 8.9% from the expected range of values, at two incidents when NN with one layer and one neuron, and an ensemble of two neural networks with only one layer and one neuron in each layer was used with a variance of 0.04 and 0.01, respectively. Figure 8a shows the comparison of predicted versus observed color values for each of the 24 harvest and location pairs for the most accurate NN implementations. In a similar fashion, Figure 8b is a visual representation of the bivariate relationship between the predicted and observed color pairs for the same implementation. It is clear that while not perfect, the color values have been predicted within a reasonable range, especially considering the wide dynamic range “a*” parameter can take, which is the indicator of redness. The highest RMSE is 4.31 and the PEP is 11.84% from the expected range of values when an ensemble of two NNs with one hidden layer and five neurons with a variance of 0.08.

**Figure 9:**
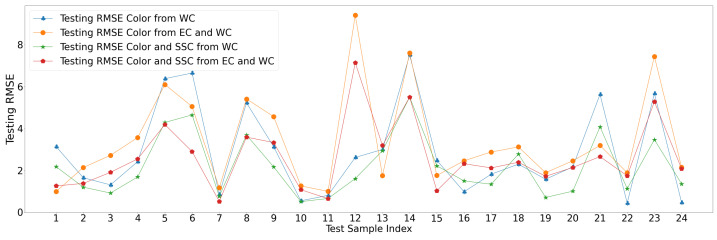
The univariate and multivariate RMSE results for GPR in predicting color, and color and SSC together from WC and WC plus EC.

**Figure 10:**
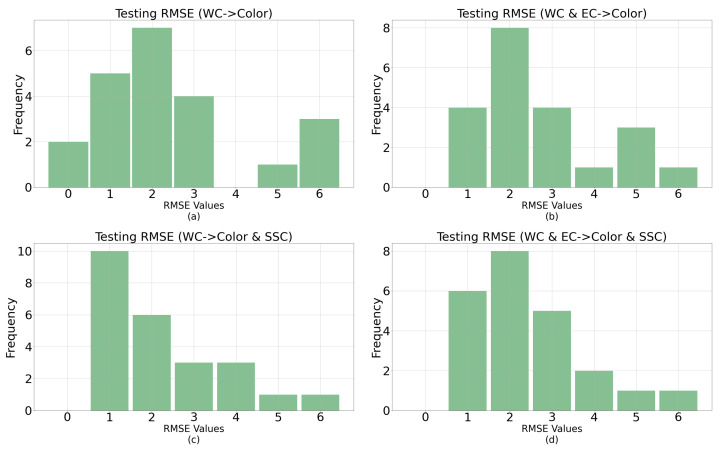
Error histograms for the four different input–output pair GPR models. (**a**) Predicting color from WC. (**b**) Predicting color from WC and EC. (**c**) Predicting color and SSC from WC. (**d**) Predicting color and SSC from WC and EC.

The GPR for the univariate response seems to have underperformed compared to the empirical methods. It is possible that the high variance in the case of the GPR could be due to the presence of some sample points, which are such outliers that they significantly impact the statistical fitting than the empirical back-propagation across the dataset [42]. Figure 9 helps visualize the impact of outlier data points, where the majority of the samples with GPR models performed well compared to the empirical models with an average RMSE below the best performing NN topology for 75% of the observations. The error distributions in Figure 10 also demonstrate that most errors occur at RMSE values of 2 or less except for the outlier points. Figure 10 shows the error histograms for each input–output pair setup where the error distributions seem mostly identical. As shown in Table 4, the average RMSE for color from WC is 3.55 on the test set, and the PEP is 9.75% with a variance of 4.43. The average RMSE for color from WC and EC is 4.09 on the test set, and the PEP is 11.25% with a variance of 5.1.

### 3.2. SSC and Color

Beyond its mathematical importance, combining the color and SSC features together can ultimately give us a more precise picture of how to adjust the color and sugar content for strawberries and improve the production based on available WC and EC averages for the 4 weeks prior to each harvest.

As anticipated with small data, the RMSE/PEP increases as the number of hidden layers and the neurons in these layers increase. However, the population of the ensemble seems to help the variance of RMSE, as shown in Figure 7b. As can be seen from Table 3, the lowest PEP is 14.04% when one NN with one layer and ten neurons was implemented with a variance of 0.03. The highest PEP is 19.04% from the expected range of values, when an ensemble of one NN with one hidden layer and two neurons with a variance of 0.12.

The GPR performed better in predicting the color values using only the WC as the input compared to using both the WC and EC as input features. Not only is the PEP% lower, but also the variance in the root mean square error is smaller. Similarly, when predicting both color and SSC, using a univariate input (WC) had a better performance than using both WC and EC as inputs, both in terms of error% (PEP) and error variance. The reader can find detailed numerical values in Table 4. This generally matches our observations using empirical models, such as the ensemble networks.

## 4. Discussion

Over the past decade, most of the research focused on evaluating the quality of specialty crops postharvest, whereas this study aims to develop a methodology to control the production quality of strawberries pre-harvest. Specifically, this paper discusses the algorithm development stage of a sensor-enabled real-time strawberry production monitoring system to improve both resource management and harvest quality. Recent studies tried to predict the crop yield [16,19,20,34] and classify the quality of the harvest based on computer-vision-developed methods using machine learning algorithms [33,43]. One of the studies by Sim et al. (2020) predicted the strawberry yield and strawberry growth stage using some environmental parameters (air temperature, soil temperature, and photosynthetic active radiation) and soil parameters (soil moisture content, EC, relative humidity, and CO_2_ concentration) [44], while Madhavi et al. [45] tried to evaluate the strawberry soil nutrition from the strawberry leaf color and predict the strawberry growth stage. Another study by ElMasry et al. [43] developed multiple linear regression models to predict the sweetness and acidity of the strawberry crop using hyper-spectral imaging in the visible and near-infrared regions.

This paper presents a nondestructive method to predict strawberry quality based on appearance (color) and flavor (sweetness) from the soil properties (WC and EC) during the pre-harvest process. Compared to the previous studies, the novel contributions of this study are multi-fold. Specifically, it introduces both empirical and statistical models to find novel correlations between the soil sensory measurements and associated multivariate harvest quality. Furthermore, it demonstrates that a sufficient prediction error can be achieved to enable a future real-time implementation of a smart controller to exploit environmental and climate conditions to reduce resource use.

Based on the structure of the data collected from a commercial farm throughout an entire Florida harvest season, several predictive models, statistical and empirical, were proposed to infer the color and sugar content of the harvested strawberries from the measured WC of the soil across a certain time period. To the best of our knowledge, both the collected data and the subsequently developed algorithms are unprecedented in the literature. This study demonstrates the performance of the algorithms through a series of use-case scenarios in predicting the univariate and multivariate responses in the form of color and sugar content of the produced strawberries as the most important physicochemical characteristics for marketability.

The average color values in the collected data were around 36.4 (a quantitative measure for redness) and the average SSC values were 6.4 (a quantitative measure of sugar content). To accurately display the performance of each prediction model, these average values are used to create the PEP metric such that the RMSE values are contrasted against the average quality values for color and SSC to provide a general understanding of the relative accuracy with respect to quantifiable metrics for each output. In the end, the models were able to predict the color and color-sugar content within as little as 9% and 14% PEP of their expected range of values, respectively. Such accuracy values allow for us to implement the next phase of the project in controlling the WC based on predicted color values once the desired quality threshold is chosen.

The authors also acknowledge the limitations of this study. Specifically, the data were collected across a single harvest season and the number of harvests for quality analysis was not enough to create a sufficiently generalizable input–output relationship for the prediction models. Even though widely accepted cross-validation methods have been deployed and validated, the study can greatly benefit from additional data points which are projected to be collected in the upcoming strawberry seasons.

These findings suggest several courses of action to implement the proposed scheme of work shown in Figure 1, as part of a large soil study in improving strawberry production using sensor-assisted real-time field monitoring. The ultimate goal of the project is to be able to control the water levels of the soil on-demand to achieve the highest possible harvest quality in terms of the physicochemical characteristics of the fruit. This study is the first step toward establishing the relationship between water content, electric conductivity, and the quality of the strawberries. A natural progression of this work is to implement one of the developed algorithms in an experimental field where water levels can be monitored and managed, and the harvest quality can be compared to the predicted algorithmic outputs. For example, the control algorithm can be tested in a real-world environment where the water levels are monitored and regulated by smart controllers at the drip line valves, maximizing resource efficiency based on dynamic environmental and weather conditions.

## 5. Conclusions

This paper presents the second stage of a system that uses sensor networks to collect real-time data for the precise monitoring and control of soil conditions in a commercial strawberry farm. The sensor network is distributed in the soil to gather data that can infer the ultimate physicochemical characteristics of the fruit at the point of harvest. Empirical and statistical models, such as neural networks and Gaussian process regression models, are used to predict the most significant physicochemical qualities of strawberry, such as color and sweetness. The level of accuracy achieved is as low as 9% and 14% of the expected range of values, respectively, which will enable the next and third phase of controlling soil conditions for sustainable and high-quality strawberry production.

This paper introduces both empirical and statistical models to discover new relationships between soil sensor measurements and multivariate harvest quality. Furthermore, it shows that it is possible to achieve a sufficient level of prediction accuracy to implement a real-time smart controller in the future to optimize resource use based on environmental and climate conditions.

The limitations of this study mainly include a lack of sufficient harvests to match the otherwise very-high-resolution multimodal data streaming from the soil sensors. This mismatch in data inputs and outputs limited the complexity of the model used for training. In future studies, more harvest points will be included and the effects of different cultivars, soil conditions, and temperatures across multiple climates will be studied through multi-climate and multiple field partnerships.

## Figures and Tables

**Table 1:** Dataset characteristics for the raw and pre-processed field and harvest data.

	Raw Dataset	Pre-Processed Dataset for Analytics
Number of soil sensor data points	22,000 per sensor	24 × 4 (WC) 24 × 8 (WC & EC)
Number of harvests for quality analysis	4	4
Number of sensors	6	6
Number of fruit samples analyzed per harvest	5–15 per sensor	Averaged across the population for each sensor

**Table 2:** Performance comparisons across all empirical models in predicting color for 50 experimental repetitions from WC, and WC plus EC, respectively.

		Color Prediction Results from WC				Color Prediction Results from WC and EC			
#Neurons	Statistical Value	Neural Network (NN)	Ensemble of 2 NN	Ensemble of 3 NN	Ensemble of 4 NN	Neural Network (NN)	Ensemble of 2 NN	Ensemble of 3 NN	Ensemble of 4 NN
[1] Neuron	Mean	3.24	3.24	3.29	3.26	4.05	4.15	4.02	4.06
	Error%	**8.90%** ^1^	**8.90%** ^1^	9.04%	8.96%	11.13%	11.40%	11.04%	11.15%
	Variance	0.04	**0.01** ^1^	**0.01** ^1^	**0.01** ^1^	0.09	0.04	0.03	0.02
[2] Neurons	Mean	3.29	3.30	3.37	3.35	4.24	4.18	4.17	4.27
	Error%	9.04%	9.07%	9.26%	9.20%	11.65%	11.48%	11.46%	11.73%
	Variance	0.05	0.02	0.04	0.24	**0.32** ^2^	0.11	0.07	0.12
[5] Neurons	Mean	3.43	3.38	3.51	3.43	4.16	4.31	4.23	4.27
	Error%	9.42%	9.29%	9.64%	9.42%	11.43%	**11.84%** ^2^	11.62%	11.73%
	Variance	0.08	0.05	0.02	0.29	0.08	0.08	0.04	0.03
[10] Neurons	Mean	3.49	3.51	3.52	3.56	4.18	4.18	4.18	4.19
	Error%	9.59%	9.64%	9.67%	9.78%	11.48%	11.48%	11.48%	11.51%
	Variance	0.08	0.06	0.05	0.25	0.09	0.04	0.04	0.02
[1, 1] Neurons	Mean	3.28	3.29	3.28	3.29	3.89	3.9	3.93	3.91
	Error%	9.01%	9.04%	9.01%	9.04%	10.69%	10.71%	10.80%	10.74%
	Variance	0.03	0.02	**0.01** ^1^	**0.01** ^1^	0.10	0.04	0.04	0.03
[2, 1] Neurons	Mean	3.28	3.27	3.30	3.25	3.97	4.02	3.95	4.07
	Error%	9.01%	8.98%	9.07%	8.93%	10.91%	11.04%	10.85%	11.18%
	Variance	0.06	0.04	0.03	0.02	0.10	0.09	0.05	0.24
[2, 2] Neurons	Mean	3.32	3.38	3.36	3.34	3.94	3.93	4.01	4.02
	Error%	9.12%	9.29%	9.23%	9.18%	10.82%	10.80%	11.02%	11.04%
	Variance	0.08	0.06	0.02	0.02	0.15	0.05	0.04	0.04
[5, 1] Neurons	Mean	3.46	3.33	3.35	3.36	4.07	4.03	4.01	4.03
	Error%	9.51%	9.15%	9.20%	9.23%	11.18%	11.07%	11.02%	11.07%
	Variance	0.09	0.04	0.24	0.03	0.16	0.04	0.04	0.04
[5, 2] Neurons	Mean	3.39	3.52	3.45	3.43	4.09	4.13	4.05	4.09
	Error%	9.31%	9.67%	9.48%	9.42%	11.24%	11.35%	11.13%	11.24%
	Variance	0.12	0.08	0.27	0.03	0.09	0.06	0.07	0.04
[5, 5] Neurons	Mean	3.49	3.49	3.52	3.54	4.11	4.16	4.09	4.11
	Error%	9.59%	9.59%	9.67%	9.73%	11.29%	11.43%	11.24%	11.29%
	Variance	0.08	0.04	0.28	0.02	0.17	0.12	0.04	0.04
[10, 1] Neurons	Mean	3.40	3.46	3.42	3.47	4.13	4.1	4.07	4.09
	Error%	9.34%	9.51%	9.40%	9.53%	11.35%	11.26%	11.18%	11.24%
	Variance	0.07	0.06	0.27	0.02	0.23	0.09	0.05	0.03
[10, 2] Neurons	Mean	3.51	3.48	3.52	3.51	4.11	4.06	4.16	4.07
	Error%	9.64%	9.56%	9.67%	9.64%	11.29%	11.15%	11.43%	11.18%
	Variance	0.11	0.04	0.28	0.02	0.18	0.05	0.07	0.03
[10, 5] Neurons	Mean	3.55	3.57	3.54	3.60	4.11	4.05	4	4.02
	Error%	9.75%	9.81%	9.73%	9.89%	11.29%	11.13%	10.99%	11.04%
	Variance	0.06	0.04	0.04	0.05	0.14	0.06	0.06	0.03
[10, 10] Neurons	Mean	3.54	3.52	3.44	3.45	4	3.93	3.91	3.94
	Error%	9.73%	9.67%	9.45%	9.48%	10.99%	10.80%	10.74%	10.82%
	Variance	0.08	0.05	0.03	0.03	0.20	0.05	0.04	0.02

^1^ The smallest error% and variance values. ^2^ The highest error% and variance values.

**Table 3:** Performance comparisons across all empirical models in predicting color and SSC for 50 experimental repetitions from WC, and WC plus EC, respectively.

		Color and SSC Prediction Results from WC				Color and SSC Prediction Results from WC and EC			
#Neurons	Statistical Value	Neural Network (NN)	Ensemble of 2 NN	Ensemble of 3 NN	Ensemble of 4 NN	Neural Network (NN)	Ensemble of 2 NN	Ensemble of 3 NN	Ensemble of 4 NN
[1] Neuron	Mean	2.61	2.63	2.61	2.57	3.09	3.09	3.08	3.09
	Error%	16.33%	16.36%	16.40%	16.39%	18.51%	18.51%	18.44%	18.52%
	Variance	0.02	**0.01** ^1^	0.14	**0.01** ^1^	0.03	0.02	**0.01** ^1^	**0.01** ^1^
[2] Neurons	Mean	2.65	2.59	2.64	2.62	3.24	3.18	3.19	3.28
	Error%	15.16%	15.35%	15.37%	15.31%	**19.04%** ^2^	18.77%	18.73%	19.02%
	Variance	0.03	**0.01** ^1^	0.15	**0.01** ^1^	0.12	0.04	0.07	**0.19** ^2^
[5] Neurons	Mean	2.64	2.62	2.63	2.65	3.04	3.05	3.08	3.06
	Error%	14.40%	14.33%	14.42%	14.31%	17.94%	17.76%	18.04%	17.87%
	Variance	0.03	**0.01** ^1^	0.16	**0.01** ^1^	0.05	0.02	0.02	**0.01** ^1^
[10] Neurons	Mean	**2.61** ^1^	2.6	2.61	2.62	2.98	3.01	3	3.01
	Error%	**14.04%** ^1^	14.11%	14.13%	14.16%	17.54%	17.54%	17.67%	17.53%
	Variance	0.03	0.03	0.15	**0.01** ^1^	0.05	**0.01** ^1^	**0.01** ^1^	**0.01** ^1^
[1, 1] Neurons	Mean	2.61	2.62	2.59	2.59	3.02	3	3.02	3.04
	Error%	16.35%	16.49%	16.45%	16.49%	17.97%	17.92%	17.90%	18.00%
	Variance	0.02	**0.01** ^1^	**0.01** ^1^	**0.01** ^1^	0.04	0.02	0.02	**0.01** ^1^
[2, 1] Neurons	Mean	2.64	2.62	2.61	2.62	3.03	2.99	2.99	3
	Error%	16.37%	16.39%	16.35%	16.53%	17.94%	17.93%	17.89%	18.00%
	Variance	0.02	**0.01** ^1^	**0.01** ^1^	**0.01** ^1^	0.06	0.02	0.02	**0.01** ^1^
[2, 2] Neurons	Mean	2.62	2.68	2.64	2.64	2.99	3	3.01	3.02
	Error%	16.15%	16.07%	15.98%	15.87%	17.97%	17.93%	17.98%	17.99%
	Variance	0.03	0.02	0.15	**0.01** ^1^	0.07	0.05	0.03	0.02
[5, 1] Neurons	Mean	2.7	2.71	2.73	2.69	3.01	3.04	3.01	3.01
	Error%	16.63%	16.33%	16.57%	15.84%	18.06%	18.15%	18.04%	18.13%
	Variance	0.04	0.02	0.02	0.02	0.05	0.04	**0.01** ^1^	0.02
[5, 2] Neurons	Mean	2.77	2.77	2.78	2.75	3.08	3.07	3.04	3.05
	Error%	15.81%	15.93%	15.72%	15.41%	18.24%	18.26%	18.01%	18.11%
	Variance	0.08	0.04	0.03	0.03	0.08	0.03	0.04	0.02
[5, 5] Neurons	Mean	2.77	2.78	2.78	2.77	3.06	3.11	3.1	3.07
	Error%	15.32%	15.52%	15.56%	15.41%	18.30%	18.20%	18.34%	18.18%
	Variance	0.05	0.03	0.04	**0.01** ^1^	0.08	0.05	0.03	0.02
[10, 1] Neurons	Mean	2.71	2.72	2.7	2.7	3.05	3	3.02	2.98
	Error%	16.63%	16.52%	16.47%	16.40%	18.23%	17.98%	18.07%	17.96%
	Variance	0.07	0.02	0.02	**0.01** ^1^	0.06	0.02	**0.01** ^1^	**0.01** ^1^
[10, 2] Neurons	Mean	2.85	2.79	2.79	2.81	3.13	3.02	3.12	3.1
	Error%	15.90%	15.80%	15.95%	15.84%	18.33%	17.90%	18.33%	18.21%
	Variance	0.04	0.05	0.03	0.02	0.09	0.03	0.08	0.02
[10, 5] Neurons	Mean	2.74	2.76	2.77	2.76	3.08	3.06	3.1	3.14
	Error%	14.97%	15.18%	15.33%	15.04%	18.05%	18.26%	18.17%	18.28%
	Variance	0.05	0.04	0.03	**0.01** ^1^	0.05	0.04	0.03	0.03
[10, 10] Neurons	Mean	2.69	2.71	2.69	2.67	3.1	3.12	3.06	3.04
	Error%	14.44%	14.51%	14.59%	14.49%	18.12%	18.26%	17.94%	17.89%
	Variance	0.04	0.03	0.02	0.02	0.08	0.05	0.02	**0.01** ^1^

^1^ The smallest error% and variance values. ^2^ The highest error% and variance values.

**Table 4:** The performance of GPR for color, color and SSC from WC and WC plus EC, respectively.

	Color Prediction from WC	Color Prediction from EC and WC	Color and SSC Prediction from WC	Color and SSC Prediction from WC and EC
Mean	3.55	4.09	2.59	3.05
Error%	9.75%	11.25%	21.15%	25.79%
Variance	4.43	5.1	1.95	2.49

## Data Availability

The data presented in this study are openly available in Mendeley repository at doi reference number 10.17632/87stbx334b.1. The used dataset and the codes for the statistical analysis can be found in the Mendeley repository.

## Abbreviations

The following abbreviations are used in this manuscript:

AI	Artificial Intelligence
DL	Deep Learning
EC	Electrical Conductivity
ENN	Ensemble of Neural Networks
GPR	Gaussian Process Regression
KNN	K-Nearest Neighbor
ML	Machine Learning
NN	Neural Networks
PEP	Prediction Error Percentage
QA	Quality Average
RMSE	Root Mean Square Error
SSC	Soluble Solids Content
SVM	Support Vector Machine
WC	Water Content
